# ECG-Free Heartbeat Detection in Seismocardiography and Gyrocardiography Signals Provides Acceptable Heart Rate Variability Indices in Healthy and Pathological Subjects

**DOI:** 10.3390/s23198114

**Published:** 2023-09-27

**Authors:** Salvatore Parlato, Jessica Centracchio, Daniele Esposito, Paolo Bifulco, Emilio Andreozzi

**Affiliations:** Department of Electrical Engineering and Information Technologies, University of Naples Federico II, Via Claudio 21, 80125 Naples, Italy; sal.parlato@studenti.unina.it (S.P.); daniele.esposito@unina.it (D.E.); paolo.bifulco@unina.it (P.B.)

**Keywords:** gyrocardiography, seismocardiography, heart rate variability, heartbeat detection, template matching, heart rate, mechanocardiography

## Abstract

Cardio-mechanical monitoring techniques, such as Seismocardiography (SCG) and Gyrocardiography (GCG), have received an ever-growing interest in recent years as potential alternatives to Electrocardiography (ECG) for heart rate monitoring. Wearable SCG and GCG devices based on lightweight accelerometers and gyroscopes are particularly appealing for continuous, long-term monitoring of heart rate and its variability (HRV). Heartbeat detection in cardio-mechanical signals is usually performed with the support of a concurrent ECG lead, which, however, limits their applicability in standalone cardio-mechanical monitoring applications. The complex and variable morphology of SCG and GCG signals makes the ECG-free heartbeat detection task quite challenging; therefore, only a few methods have been proposed. Very recently, a template matching method based on normalized cross-correlation (NCC) has been demonstrated to provide very accurate detection of heartbeats and estimation of inter-beat intervals in SCG and GCG signals of pathological subjects. In this study, the accuracy of HRV indices obtained with this template matching method is evaluated by comparison with ECG. Tests were performed on two public datasets of SCG and GCG signals from healthy and pathological subjects. Linear regression, correlation, and Bland-Altman analyses were carried out to evaluate the agreement of 24 HRV indices obtained from SCG and GCG signals with those obtained from ECG signals, simultaneously acquired from the same subjects. The results of this study show that the NCC-based template matching method allowed estimating HRV indices from SCG and GCG signals of healthy subjects with acceptable accuracy. On healthy subjects, the relative errors on time-domain indices ranged from 0.25% to 15%, on frequency-domain indices ranged from 10% to 20%, and on non-linear indices were within 8%. The estimates obtained on signals from pathological subjects were affected by larger errors. Overall, GCG provided slightly better performances as compared to SCG, both on healthy and pathological subjects. These findings provide, for the first time, clear evidence that monitoring HRV via SCG and GCG sensors without concurrent ECG is feasible with the NCC-based template matching method for heartbeat detection.

## 1. Introduction

Heart rate variability (HRV) is defined as the beat-to-beat variation in heart rate or inter-beat interval due to the functioning of the autonomic nervous system under physiological mechanisms, mainly respiration, baroreflexes, and thermoregulation [1,2,3,4,5,6]. In healthy people, HRV exhibits repeating patterns with the circadian rhythms. On the other hand, the alteration or loss of this variability has prognostic significance in several pathological conditions. The analysis of HRV has been recognized as a valuable diagnostic tool as it provides early warning signs for autonomic impairments in many life-threatening cardiac and non-cardiac diseases [1,2,3,4,5,6]. HRV analysis is a critical task in the monitoring of people at high risk for sudden cardiac death, such as post-infarction survivors, individuals affected by congestive heart failure, valvular dysfunctions or cardiomyopathies, heart transplantation recipients, or subjects with a history of myocardial ischemia [1,2,3,4,5,6,7,8,9,10]. Abnormalities in the physiological modulation of heart rhythm are also associated with different non-cardiac diseases. These include diabetes mellitus, obesity, end-stage renal failure, stroke, multiple sclerosis, muscular dystrophy, epilepsy, and neonatal distress syndrome [1,2,3,4,5,6,10,11]. Moreover, HRV indices are useful to assess mental health and help in the diagnosis of emotional disorders [10,12]. HRV analysis has also been investigated in sport science and rehabilitation medicine to monitor the autonomic response to physical exercise [10,13,14]. Furthermore, the evaluation of HRV has an important role in promoting people’s well-being since it is affected by many lifestyle factors, such as sleep, drugs, stress, diet, alcohol consumption, smoking, etc. [2,10,15,16]. HRV is also used in sleep studies [17], e.g., to support the sleep staging task [18]. 

Different approaches are available for HRV analysis. They can be essentially divided into three classes: time-domain methods, frequency-domain methods, and non-linear methods. In time-domain HRV analysis, many statistical and geometric indices are computed from the time series of inter-beat intervals, also known as the tachogram. On the contrary, various spectral analyses are applied to the tachogram to obtain HRV measures in the frequency domain. Furthermore, non-linear parameters are often evaluated from the inter-beat intervals to capture HRV non-linear properties. Generally, HRV analysis is carried out on Electrocardiography (ECG) recordings acquired within a few minutes or 24 h. For this reason, a distinction can be made between short-term and long-term HRV analysis [3,4,5,19]. Heartbeat localization is a fundamental step in HRV analysis. The peaks of R waves in any ECG tracing are the most accepted fiducial points for heartbeats. Hence, inter-beat intervals are computed as temporal differences between consecutive R-peaks. ECG is certainly the reference technique in clinical practice for monitoring heart rate and, therefore, for analyzing its variability [1,2,3,4,5,6]. However, it requires supervision by trained clinical staff. Moreover, some practical issues must be considered: the need for stable electrodes placement over time, which could be compromised by their possible detachment or slipping, and proper skin preparation via the application of an electrolytic gel, which dries after prolonged time. In addition, the inconvenience of cable connections, the inherent susceptibility to electromagnetic interference, and electrical risks represent further drawbacks of this technique. For these reasons, ECG is not well suited to continuous, long-term monitoring, especially in non-clinical settings [20,21,22,23,24,25]. Photoplethysmography has also been investigated as a wearable surrogate for ECG in HRV analysis [26,27,28,29,30,31,32], also by using modern smart watches and with the support of machine learning [33,34,35]. However, the strong sensitivity to motion artifacts prevents precise identification of heartbeat markers, thus limiting its application to continuous heart rate monitoring and therefore the possibility of obtaining accurate HRV measurements [36,37,38,39]. 

In the last decades, the great advances in sensor technology have led to a growing interest in cardio-mechanical monitoring techniques, which monitor the small vibrations induced on the body surface by the mechanical activity of the cardiovascular system. In addition to the well-established Phonocardiography (PCG) [40,41,42,43], alternative techniques such as Ballistocardiography (BCG) [44,45,46,47,48,49] and Seismocardiography (SCG) [50,51,52,53,54,55,56,57] have regained attention due to their suitability for wearable applications. Novel techniques have also been introduced, such as Gyrocardiography (GCG) [58,59,60,61], Kinocardiography (KCG) [62,63] and Forcecardiography (FCG) [64,65,66,67,68,69,70,71]. BCG signals are commonly measured via weighing scales as well as sensing systems embedded in beds or chairs to capture the mechanical vibrations of the whole body due to blood flowing through the vascular tree. On the other hand, SCG and GCG make use of three-dimensional accelerometers and gyroscopes to record linear accelerations and angular velocities of the chest wall, respectively. Combined SCG and GCG measurements have also been investigated thanks to the availability of inertial measurement units (IMUs) that integrate both types of sensors [72]. KCG uses two IMUs, placed on the chest and on the lower back, thus performing 12 degrees-of-freedom measurements. Finally, FCG captures respiration, infrasonic cardiac vibrations, and heart sounds all simultaneously from a single site of the chest via broadband force sensors. 

Cardio-mechanical monitoring techniques are not affected by the typical ECG drawbacks related to electrodes. However, they are still affected by motion artifacts, which currently limit their use in long-term monitoring for clinical purposes. These techniques rely on small, lightweight, low-cost sensors, which are particularly appealing for wearable applications. In this regard, continuous, long-term monitoring would benefit from wearable devices, especially in daily life environments, all the more so if one considers that accelerometers and gyroscopes are largely integrated into smartphones nowadays. For these reasons, many studies investigated the suitability of cardio-mechanical signals for HRV analysis, particularly BCG [73,74,75], SCG [36,57,76,77,78], and GCG [76,79]. Specific peaks or valleys of these signals, which correlate with important cardiac cycle events, were considered as heartbeat fiducial points, e.g., the aortic valve opening (AO) on SCG/GCG signals and the peak of the J wave on BCG signals. However, the localization of fiducial points is usually performed by taking advantage of a simultaneous ECG recording [48,50,51,52,60], which limits the application of such methods to standalone cardio-mechanical monitoring. There were also some attempts to perform HRV analysis on time series of inter-beat intervals obtained without the support of concurrent ECG tracings [78,80,81,82,83,84,85,86]; however, these methods were only tested on limited cohorts of healthy subjects. Furthermore, it should be underlined that the cardio-mechanical signals of pathological subjects show atypical waveforms with high morphological variability [87]. This aspect makes heartbeat detection a much more complex task. Few studies by Sieciński et al. investigated the feasibility of HRV analysis on cardio-mechanical signals of pathological subjects [88,89,90]. Specifically, HRV indices obtained from the SCG and/or GCG signals of 30 healthy volunteers and 30 patients with valvular heart diseases (VHDs) were compared with those computed from reference ECG signals. The inter-beat intervals considered for HRV analysis were estimated as temporal differences between consecutive AO events, which were identified by taking advantage of simultaneous ECG signals. 

Recently, a template matching algorithm for ECG-free heartbeat detection has been presented. In detail, this method assesses the morphological similarity between a heartbeat template and the whole signal by using the normalized cross-correlation (NCC) as a similarity measure. In particular, the local maxima of the NCC function, which correspond to the signal chunks with the highest local similarity to the selected template, are considered heartbeat fiducial points. This ECG-free heartbeat detection method was tested on both SCG and GCG signals of 100 patients with one or more VHDs [91,92], made available in a public database [93]. The results of these studies demonstrated that the template matching algorithm provides high accuracy in heartbeat detection and inter-beat interval estimation, both in SCG and GCG signals. It is worth highlighting that in [91,92] the inter-beat intervals were computed from NCC local maxima instead of common SCG/GCG fiducial points, and only the inter-beat intervals related to correctly detected heartbeats in SCG and GCG signals were compared against those obtained from ECG. However, in real standalone cardio-mechanical monitoring, it is not possible to exclude inter-beat intervals related to missed and/or spurious heartbeats; therefore, their impact on HRV analysis should be evaluated. In addition, it is important to evaluate how measurement errors, although very small, affect the accuracy of HRV analysis. 

The present study addressed this issue by performing extensive HRV analyses on two public databases, consisting of SCG, GCG, and ECG recordings from 100 VHD patients [93] and 29 healthy subjects [94]. To this end, the ECG-free heartbeat detection method based on template matching was applied to these signals for inter-beat interval estimation. Then, several time-domain, frequency-domain, and non-linear HRV indices were computed from SCG and GCG data and compared with those obtained from ECG via linear regression, correlation, and Bland-Altman analyses. The results demonstrate that the HRV indices obtained by using the ECG-free heartbeat detection based on template matching are in very close agreement with those provided by ECG. 

## 2. Materials and Methods

### 2.1. Datasets

#### 2.1.1. Pathological Subjects Dataset

Two public datasets were considered for this study. The first one, which is thoroughly described in [93], consists of SCG, GCG, and ECG signals collected from 100 patients (59 males and 41 females, age 68 ± 14 years) suffering from one or more valvular heart diseases (VHDs) at a moderate or severe stage. All signals were recorded via an ECG front-end and an inertial measurement unit consisting of a tri-axial MEMS accelerometer and gyroscope, all integrated in the Shimmer 3 ECG module (Shimmer Sensing, Dublin, Ireland), which was secured on subjects’ chests. The recordings were carried out on patients lying supine and breathing at a natural pace. 

ECG leads II, SCGz, and GCGy signals from this database have already been processed in previous studies [91,92], which focused on detecting heartbeats via a novel ECG-free detection method, which is also briefly described in the next section. Some patients’ recordings have been excluded from SCG and/or GCG analysis due to extremely poor signal quality (detailed information is reported in [91,92]). The results obtained from [91,92] were used in this study for HRV analysis. 

#### 2.1.2. Healthy Subjects Dataset

The second public dataset considered in this study consists of simultaneously acquired ECG, SCG, and GCG signals, collected from 29 male healthy subjects (age 29 ± 5 years) [94]. A triaxial capacitive digital accelerometer (MMA8451Q, Freescale Semiconductor, Austin, TX, USA), a triaxial gyroscope (MAX21000, Maxim Integrated, San Jose, CA, USA), and an ECG front-end (ADS1293, Texas Instruments, Dallas, TX, USA) were used to acquire, respectively, SCG, GCG, and ECG signals, simultaneously sampled at 800 Hz. Recordings were performed with sensors attached via double-sided tape to the sternum of the subjects while lying either in the supine position or on their left or right side. 

Heartbeat detection was performed on ECG, SCG, and GCG signals from the healthy subjects database via the same procedure adopted for the pathological subjects dataset. Then, inter-beat intervals were computed and used for HRV analysis.

### 2.2. Heartbeat Detection and Inter-Beat Intervals Estimation

Inter-beat intervals used for HRV analysis were obtained from the results of previous studies [91,92]. In these studies, after a first pre-processing step aimed at resampling ECG, SCG, and GCG signals at 1 kHz via linear interpolation and improving their signal-to-noise ratio, the heartbeats were detected in such signals and eventually used to compute the inter-beat intervals. To this end, the heartbeats were detected in ECG signals by locating the R-peaks via the well-known Pan and Tompkins algorithm [95], implemented in the BioSigKit Matlab® toolbox [96]. On the other hand, the heartbeats were detected in SCG and GCG signals by using a novel ECG-free heartbeat detection algorithm based on template matching via normalized cross-correlation (NCC), which is extensively described in [91,92]. Essentially, the template matching method computes the NCC between the whole signal (SCG or GCG) and a heartbeat template selected from the same signal and then locates the local maxima of the NCC function corresponding to the time locations at which the signal exhibits the local highest similarity with the selected heartbeat template. Therefore, unlike common approaches that use specific fiducial points, such as the aortic valve opening, to locate the heartbeats in SCG and GCG signals [48,50,51,52,60], the template matching approach adopted in [91,92] provides the timings of NCC local maxima as the time locations of the heartbeats. In [91,92], only the inter-beat intervals related to correctly detected heartbeats in SCG and GCG signals were compared against those obtained from ECG, demonstrating a very close agreement. In this study, instead, the inter-beat intervals related to missed and false heartbeats were also considered in order to effectively evaluate the agreement of HRV indices obtained from SCG and GCG signals in a full standalone operation with those obtained from reference ECG. 

The same procedure was applied to the signals collected in the healthy subjects dataset. Therefore, after resampling and filtering operations, heartbeats were first detected in the ECG signals via the Pan and Tompkins algorithm and in the SCGz and GCGy signals via the ECG-free heartbeat detection algorithm based on template matching; then, the inter-beat intervals were computed from the heartbeat locations just obtained and fed to the subsequent HRV analysis. 

### 2.3. Heart Rate Variability Analysis

HRV analysis was carried out using the well-established software “*Kubios HRV Standard*”, which has been developed for more than 20 years and is enriched with state-of-the-art methodologies for accurate HRV indices extraction [97,98,99,100]. For this reason, *Kubios* has been used in many scientific studies [101,102,103,104,105,106,107,108,109]. In this study, time-domain, frequency-domain, and non-linear HRV indices were considered, which are outlined in Table 1. 

Before performing the HRV analysis, a thorough examination of ECG signals from the pathological subjects dataset was carried out to recognize all subjects with signs of atrial fibrillation/flutter or other forms of arrhythmias, as well as those presenting ECG signals where P-waves cannot be clearly identified and/or an excessive number of premature ventricular contractions occurred. This step was instrumental in excluding those subjects from HRV analysis since those anomalies in ECG signals are not related to the fluctuations of the sinus rhythm caused by autonomic nervous system control, which is the main subject of HRV analysis. 

The time series of the inter-beat intervals (i.e., tachograms) extracted from each of the ECG, SCG, and GCG signals of all subjects included in the analysis were imported into *Kubios HRV Standard*. Before actually extracting the HRV indices outlined in Table 1, each tachogram was analyzed to detect and correct possible artifacts, as recommended in the *Kubios HRV* guidelines [110]. *Kubios HRV Standard* offers a correction algorithm based on predefined thresholds (ranging from “*very low*” to “*very strong*”) or possibly a custom threshold, which are used to detect potential artifacts. A sample of the tachogram (i.e., an inter-beat interval) is classified as an artifact if the difference with a local median of the tachogram exceeds the selected threshold [110]. The detected artifacts are then corrected with values obtained via interpolation with cubic splines. The predefined threshold values decrease from the “*very low*” option to the “*very strong*” option; thus, the “*very low*” option detects only the tachogram points featuring the highest differences with the local median, while the other options toward “*very strong*” detect points with progressively lower differences. This implies that selecting a stronger correction option increases the chance of erroneously classifying a correct inter-beat interval as an artifact, which would be then replaced by an interpolated value, thus corrupting the actual HRV trend. For this reason, the *Kubios HRV* guidelines recommend that the user select the lowest correction option that allows detecting and correcting all artifacts while minimizing the number of correct points that are erroneously considered artifacts. The correction of the tachograms analyzed in this study was performed according to *Kubios HRV* guidelines. 

### 2.4. Statistical Analyses

Correlation, Passing-Bablok linear regression [111], and Bland-Altman analyses [112,113] were carried out to compare the HRV indices obtained from SCG and GCG signals with respect to those obtained from ECG, which were considered the ground truth. In particular, data from healthy and pathological subjects were analyzed separately. Therefore, for each HRV index, the values obtained from SCG signals of all subjects in the considered group were compared with those obtained from the related ECG signals; the same procedure was then applied to the values obtained from GCG signals. Correlation and Bland-Altman analyses were performed via the Matlab® function *bland-altman-and-correlation-plot* [114]. 

## 3. Results

### 3.1. Analysis of Healthy Subjects Data

All 29 subjects in the healthy subjects dataset were considered for the analyses, which were performed on a total of 10,967 inter-beat intervals. The results of linear regression and correlation analyses and of Bland-Altman analysis performed on HRV indices extracted from SCG signals are outlined in Table 2 and Table 3, respectively, while those related to GCG signals are outlined in Table 4 and Table 5. 

The results of correlation analyses report that all HRV indices extracted from both SCG and GCG achieved Pearson’s correlation coefficients (with respect to ECG) in excess of 0.9, apart from *Sample entropy* obtained from GCG. However, the 95% confidence intervals (CI) of correlation coefficients of some HRV indices presented lower bounds below 0.9, namely, *Max HR* and *Sample entropy* estimated from SCG and GCG. According to the results of Passing-Bablok linear regression, for all HRV indices extracted from SCG, no statistically significant differences from unity were found for the slopes (i.e., the CIs of the slopes always included unity), while statistically significant intercepts were found for *Mean RR*, *Mean HR*, *LF absolute power*, and *Poincaré SD2*. Concerning the results achieved on GCG signals, no statistically significant differences from unity were found for the slopes of all HRV indices but the RMSSD, while statistically significant intercepts were found for *SDNN*, *SD HR*, *Min HR*, *RMSSD*, *Poincaré SD1*, and *Poincaré SD2*. In addition, the cusum test [111] confirmed the linearity between all HRV indices extracted from SCG and ECG signals (*p* > 0.1) and from GCG and ECG signals (*p* > 0.1). The results of Bland-Altman analyses revealed modest yet statistically significant biases for *SDNN*, *SD HR*, *Min HR*, *LF absolute power*, *HF absolute power*, and *Total power* estimated from both SCG and GCG signals, as well as for *Max HR*, *Poincaré SD2*, and *DFA α2* estimated from SCG, and *RMSSD*, *NN50*, *pNN50*, and *LF relative power* estimated from GCG.

**Figure 1:**
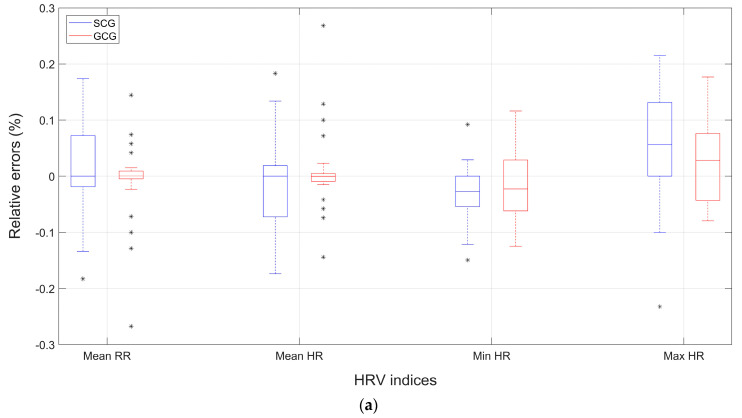

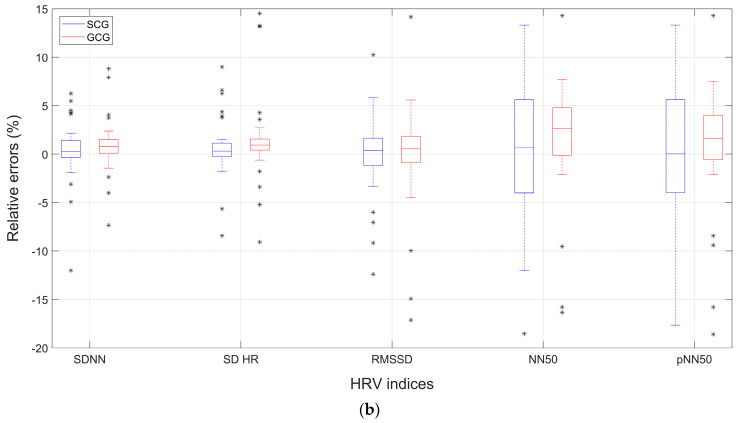

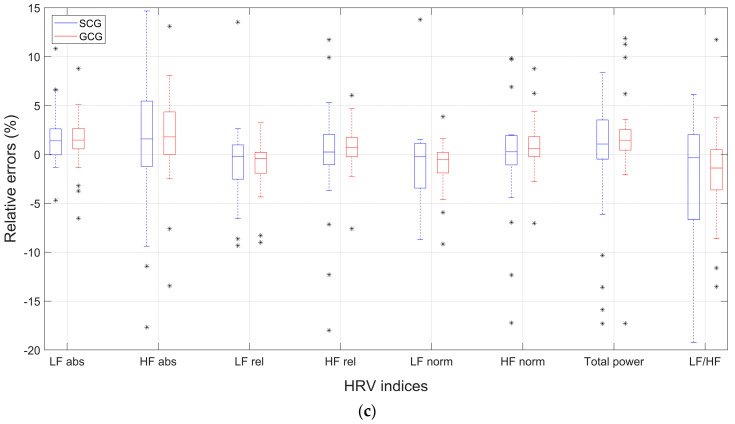

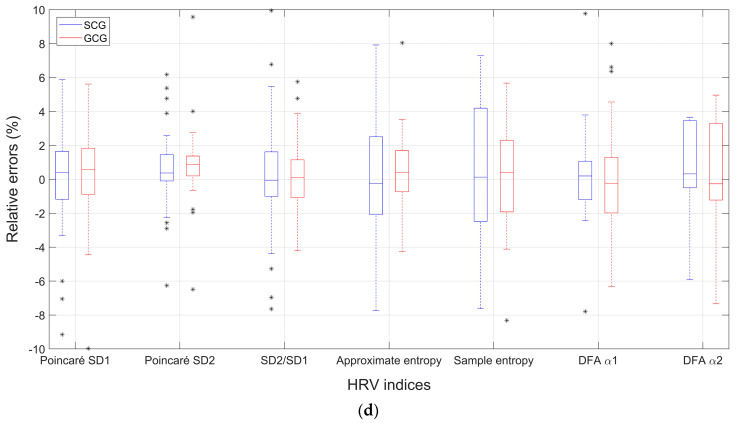
Comparison of box and whiskers plots of relative percentage errors of HRV indices extracted from SCG and GCG signals of the healthy subjects dataset: (**a**,**b**) time-domain indices; (**c**) I frequency-domain indices; (**d**) non-linear indices.

Figure 1 shows the box and whiskers plots of the relative percentage errors of HRV indices obtained from SCG and GCG. In particular, the results related to time-domain indices are shown in panels (Figure 1a,b), those related to frequency-domain indices are shown in panel (Figure 1c), and those related to non-linear indices are shown in panel (Figure 1d). 

### 3.2. Analysis of Pathological Subjects Data

A total of 51 pathological subjects were considered for the analyses (see Table A1 in Appendix A). Six subjects were excluded from the SCG data analysis and two subjects from the GCG data analysis because of the poor signal quality [91,92]. Therefore, HRV indices were extracted from SCG signals of 45 subjects (total of 18,429 inter-beat intervals) and GCG signals of 49 subjects (total of 20,578 inter-beat intervals). The results of linear regression and correlation analyses and of Bland-Altman analysis performed on HRV indices extracted from SCG signals are outlined in Table 6 and Table 7, respectively. The results obtained on HRV indices extracted from GCG signals are outlined in Table 8 and Table 9. 

The results of correlation analyses report that all HRV indices extracted from both SCG and GCG achieved Pearson’s correlation coefficients (with respect to ECG) in excess of 0.9, apart from *LF/HF* and *Sample entropy* obtained from SCG and GCG, as well as *LF absolute, relative, and normalized powers*, *HF relative and normalized powers*, *SD2/SD1*, and *DFA α1* obtained from GCG. However, the correlation coefficients Cis of some HRV indices presented lower bounds below 0.9, namely, *LF relative power*, *HF relative power*, *LF normalized power*, *HF normalized power*, *LF/HF*, *SD2/SD1*, *Sample entropy*, and *DFA α1* estimated from SCG and GCG, along with *Total power* estimated from SCG and *LF absolute power*, *HF absolute power*, *and Approximate entropy* obtained from GCG. According to the results of Passing-Bablok linear regression, no statistically significant differences from unity were found for the slopes of all HRV indices, apart from *SDNN*, *RMSSD*, and *HF absolute power* obtained from SCG and GCG, as well as *Max HR*, *NN50*, *pNN50*, and *Poincaré SD2* estimated from SCG and *SD HR*, *Total power*, *Poincaré SD1*, and *DFA α2* estimated from GCG. Statistically significant intercepts were found for *Mean RR*, *SDNN*, *Mean HR*, *Max HR*, *RMSSD*, *HF absolute power*, *Poincaré SD1*, and *Poincaré SD2* obtained from SCG and for *SDNN*, *SD HR*, *RMSSD*, *HF absolute power*, *Total power*, *Poincaré SD1*, and *DFA α2* estimated from GCG. In addition, the cusum test [111] confirmed the linearity between all HRV indices extracted from SCG and ECG signals (*p* > 0.05 for *Max HR* and *p* > 0.1 for all other indices) and from GCG and ECG signals (*p* > 0.1). The results of Bland-Altman analyses revealed modest yet statistically significant biases for *Mean RR*, *Mean HR*, *Min HR, LF absolute power*, *and HF absolute power* estimated from both SCG and GCG signals, as well as for *LF relative power*, *HF relative power*, *LF normalized power*, *HF normalized power*, *LF/HF*, and *SD2/SD1* obtained from GCG. 

**Figure 2:**
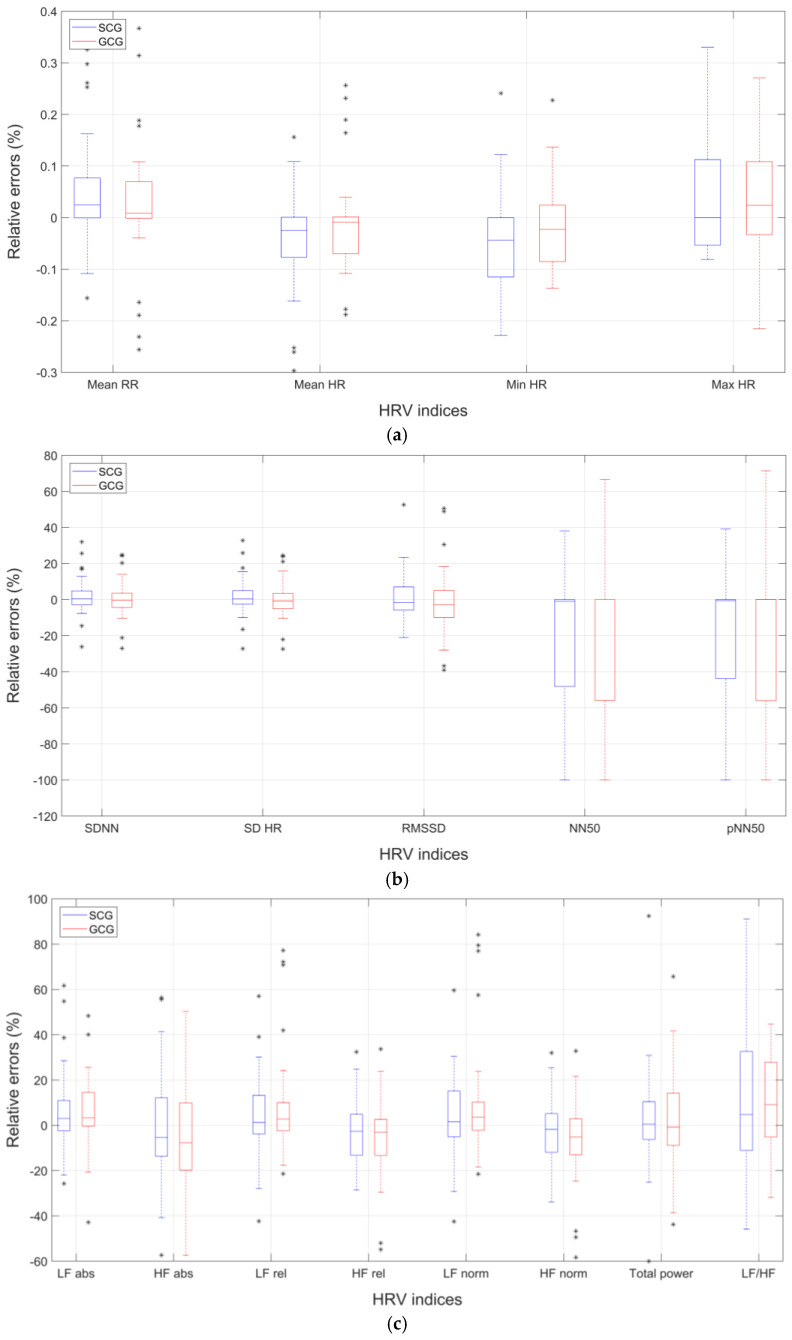

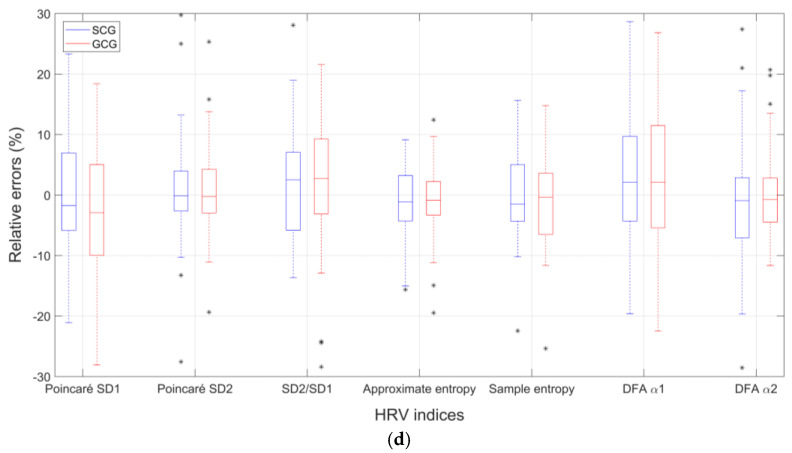
Comparison of box and whiskers plots of relative percentage errors of HRV indices extracted from SCG and GCG signals of the pathological subjects dataset: (**a**,**b**) time-domain indices; (**c**) I frequency-domain indices; (**d**) non-linear indices.

Figure 2 shows the box and whiskers plots of the relative percentage errors of HRV indices obtained from SCG and GCG signals of the 40 subjects for which both SCG and GCG analyses were performed. In particular, the results related to time-domain indices are shown in panels (Figure 2a,b), those related to frequency-domain indices are shown in panel (Figure 2c), and those related to non-linear indices are shown in panel (Figure 2d). 

## 4. Discussion

This study evaluated the accuracy of HRV indices computed from inter-beat intervals estimated from SCG and GCG signals via an ECG-free heartbeat detection algorithm based on template matching. The inter-beat intervals were computed from heartbeat locations determined as local maxima of the normalized cross-correlation between the whole signal and a heartbeat template selected from the same signal. In principle, the inter-beat intervals thus obtained are affected by two error contributions: (1) missed and spurious heartbeats, which introduce false increases and decreases in inter-beat intervals; and (2) the potential inconstancy of the locations of NCC maxima within successive cardiac cycles, which introduces inaccuracies in inter-beat interval estimation. 

The results of this study show that the ECG-free heartbeat detection algorithm based on template matching allowed estimating HRV indices from SCG and GCG signals of healthy subjects with acceptable accuracy. The estimates obtained on signals from pathological subjects were affected by larger errors. In particular, errors achieved in healthy subjects for *Mean RR*, *Mean HR*, *Min HR*, and *Max HR* turned out to be confined within 0.25%; *SDNN* and *SD HR* within 2%; *RMSSD* within 5%; *NN50* and *pNN50* within 15%; *HF absolute power* and *LF/HF* within 20%; all other frequency-domain indices within 10%; and non-linear domain indices within 8%. For pathological subjects, errors on *Mean RR*, *Mean HR*, *Min HR*, and *Max HR* were confined within 0.35%; *SDNN* and *SD HR* within 15%; *RMSSD* within 25%; *NN50* and *pNN50* within 100%; *HF absolute power* and *LF/HF* within 90%; all other frequency-domain indices within 40%; and non-linear indices within 30%. Overall, GCG provided slightly better or at least comparable performances with respect to SCG, both on healthy and pathological subjects. 

Only a few studies in the literature have evaluated the accuracy of HRV indices extracted from SCG and GCG signals. Some of these studies performed heartbeat detection by taking advantage of a concurrently acquired ECG lead; this aspect limits the significance of their results for standalone cardio-mechanical monitoring. In addition, all methods presented in the literature are based on the localization of specific fiducial points in SCG and GCG signals, which represents a potential weakness. Indeed, according to the findings of [87], many pathological subjects exhibit signal morphologies that do not allow clear identification of fiducial points. Hence, in such cases, the heartbeat detection methods based on fiducial points could fail or at least provide inaccurate measures of inter-beat intervals. This study demonstrated that acceptable HRV indices can be computed from NCC local maxima provided by the ECG-free heartbeat detection method based on template matching, which overcomes potential issues in localizing SCG/GCG fiducial points. It is also worth underlining that only [88,89,90] analyzed signals from pathological subjects, while all other studies focused on small cohorts of healthy subjects. In addition, many of these studies do not report the number of inter-beat intervals considered for HRV analysis, which impacts the reliability of the statistical analyses. Unfortunately, none of these studies presented a thorough evaluation of the agreement between HRV indices obtained from SCG/GCG and ECG by using linear regression and Bland-Altman analyses. Some studies performed Student *t*-tests to verify, at least, if differences in HRV indices were statistically significant. In some cases, a Bland-Altman comparison between inter-beat intervals obtained from SCG/GCG signals and from ECG was performed; however, reporting limits of agreement considerably higher than those achieved by the ECG-free heartbeat detection method adopted in this study. Such a higher LoA could explain the larger errors achieved in HRV analysis as compared to the results of this study. 

The significance of the encouraging results of this study is limited to short-term HRV indices evaluated on healthy subjects and VHD patients at rest. These limitations arise from the specific protocols adopted in data collection for the considered datasets. Further evaluations should be carried out on much longer recordings to allow long-term HRV analyses, possibly including motion artifacts due to physical activities (e.g., walking, running). Moreover, additional tests should be conducted on the cardio-mechanical signals of patients affected by other cardiac pathologies. 

## 5. Conclusions

The objective of this study was to quantitatively assess the accuracy of Heart Rate Variability indices computed from heartbeat locations identified in Seismocardiography and Gyrocardiography signals via a novel ECG-free template matching method, particularly by considering the local maxima of the normalized cross-correlation between a heartbeat template and the whole signal as heartbeat markers in place of the most commonly used SCG/GCG fiducial points (e.g., aortic valve opening). This study presents, for the first time, a thorough evaluation of the accuracy of HRV indices computed from SCG and GCG signals by considering the largest cohort of subjects among all studies presented in the literature, with the highest number of pathological subjects, and also by performing rigorous statistical analyses to assess the suitability of SCG and GCG signals for HRV analysis based on ECG-free, cardio-mechanical monitoring. 

The results demonstrate that the ECG-free template matching method for heartbeat detection provides acceptable heartbeat timings in SCG and GCG signals, so that HRV indices extracted from these signals are in very close agreement with those provided by ECG. These findings provide, for the first time, clear evidence that monitoring HRV via SCG and GCG sensors without concurrent ECG is feasible. 

## Figures and Tables

**Table 1:** Time-domain, frequency-domain, and non-linear HRV indices considered in this study.

*Time-Domain Indices*	*Frequency-Domain Indices*	*Non-Linear Indices*
Mean RR (ms)	LF absolute power (ms^2^)	Poincaré SD1 (ms)
SDNN (ms)	HF absolute power (ms^2^)	Poincaré SD2 (ms)
Mean HR (bpm)	LF relative power	Poincaré SD2/SD1
SD HR (bpm)	HF relative power	Approximate entropy
Min HR (bpm)	LF normalized power	Sample entropy
Max HR (bpm)	HF normalized power	DFA α1
RMSSD (ms)	Total power (ms^2^)	DFA α2
NN50 (beats)	LF/HF	
pNN50 (adim)		

**Table 2:** Results of correlation and Passing-Bablok linear regression analyses performed on SCG and ECG signals from the 29 subjects in the healthy subjects dataset.

*HRV Index*	*r*	*CI_r_*	*Slope*	*CI_slope_*	*Intercept*	*CI_intercept_*
Mean RR *	0.998	[0.995; 0.999]	1.003	[1.000; 1.013]	−2.840	[−12.394; −0.116]
SDNN *	0.994	[0.987; 0.997]	0.996	[0.967; 1.015]	0.343	[−0.463; 1.434]
Mean HR *	0.999	[0.997; 0.999]	1.003	[1.000; 1.012]	−0.173	[−0.781; −0.008]
SD HR *	0.982	[0.962; 0.992]	0.991	[0.939; 1.011]	0.042	[−0.022; 0.188]
Min HR *	0.982	[0.962; 0.992]	1.000	[0.999; 1.002]	0.009	[−0.117; 0.066]
Max HR *	0.915	[0.825; 0.960]	1.001	[0.996; 1.006]	−0.037	[−0.418; 0.371]
RMSSD *	0.993	[0.985; 0.997]	0.998	[0.969; 1.022]	0.313	[−0.778; 1.388]
NN50 *	0.998	[0.996; 0.999]	1.000	[0.972; 1.031]	1.000	[−3.047; 2.593]
pNN50 *	0.997	[0.994; 0.999]	1.012	[0.975; 1.052]	−0.211	[−1.072; 0.838]
LF absolute power *	0.984	[0.967; 0.993]	0.998	[0.973; 1.012]	10.921	[0.503; 37.109]
HF absolute power *	0.983	[0.965; 0.992]	1.004	[0.959; 1.032]	9.575	[−11.543; 47.347]
LF relative power *	0.974	[0.945; 0.988]	0.990	[0.930; 1.018]	0.475	[−1.152; 3.107]
HF relative power *	0.974	[0.945; 0.988]	0.991	[0.932; 1.021]	0.506	[−0.883; 3.799]
LF normalized power *	0.974	[0.945; 0.988]	0.991	[0.932; 1.022]	0.421	[−1.194; 2.741]
HF normalized power *	0.974	[0.945; 0.988]	0.991	[0.931; 1.022]	0.503	[−0.975; 4.071]
Total power *	0.993	[0.985; 0.997]	0.995	[0.936; 1.014]	26.638	[−6.793; 115.472]
LF/HF *	0.982	[0.961; 0.991]	0.984	[0.897; 1.020]	0.012	[−0.021; 0.062]
Poincaré SD1 *	0.993	[0.985; 0.997]	0.998	[0.969; 1.021]	0.222	[−0.522; 0.992]
Poincaré SD2 *	0.995	[0.988; 0.998]	0.988	[0.957; 1.002]	0.882	[0.093; 2.399]
SD2/SD1 *	0.990	[0.979; 0.995]	0.996	[0.961; 1.042]	0.007	[−0.078; 0.066]
Approximate entropy *	0.993	[0.984; 0.997]	1.016	[0.968; 1.066]	−0.016	[−0.067; 0.029]
Sample entropy *	0.852	[0.705; 0.928]	1.025	[0.899; 1.149]	−0.036	[−0.226; 0.159]
DFA α1 *	0.972	[0.940; 0.987]	1.001	[0.971; 1.051]	0	[−0.049; 0.028]
DFA α2 *	0.967	[0.930; 0.985]	0.990	[0.925; 1.024]	0.003	[−0.003; 0.018]

* cusum linearity test *p*-value > 0.1.

**Table 3:** Results of the Bland-Altman analysis performed on SCG and ECG signals from the 29 subjects in the healthy subjects dataset In cases of measurement differences with a non-normal distribution, the bias was estimated as the median of differences and the limits of agreement as the 2.5th and 97.5th percentiles, respectively.

*HRV Index*	*Bias*	*CI_bias_*	*LoA*	*CI_LoA min_*	*CI_LoA max_*
Mean RR *	0	[−0.061; 0.030]	[−3.319; 33.299]	[−3.365; −2.619]	[25.352; 35.487]
SDNN *	0.106	[0.069; 0.478]	[−11.024; 3.284]	[−12.528; −4.828]	[2.841; 3.379]
Mean HR *	0	[−0.002; 0.007]	[−1.616; 0.213]	[−1.623; −1.177]	[0.158; 0.224]
SD HR *	0.013	[0.002; 0.026]	[−1.032; 0.233]	[−1.204; −0.354]	[0.202; 0.234]
Min HR *	−0.018	[−0.028; −0.012]	[−5.775; 1.451]	[−7.080; −1.015]	[0.043; 1.857]
Max HR *	0.038	[0.019; 0.060]	[−15.102; 5.219]	[−15.254; −11.316]	[1.213; 6.310]
RMSSD *	0.239	[−0.03; 0.403]	[−13.254; 4.288]	[−14.591; −7.061]	[3.274; 4.456]
NN50	−0.138	[−2.025; 1.75]	[−10.476; 10.200]	[−13.838; −7.114]	[6.838; 13.563]
pNN50	0.136	[−0.441; 0.712]	[−3.021; 3.292]	[−4.048; −1.995]	[2.266; 4.319]
LF absolute power *	8.032	[3.756; 22.21]	[−1991.966; 523.142]	[−2262.832; −841.009]	[69.770; 653.836]
HF absolute power *	12.248	[0.77; 27.906]	[−958.158; 489.074]	[−1132.237; −306.394]	[219.932; 558.145]
LF relative power *	−0.063	[−0.781; 0.174]	[−12.024; 9.500]	[−14.202; −4.461]	[6.477; 9.983]
HF relative power *	0.067	[−0.284; 0.373]	[−9.651; 12.934]	[−9.930; −6.902]	[4.421; 15.369]
LF normalized power *	−0.078	[−0.407; 0.303]	[−13.007; 9.697]	[−15.444; −4.456]	[6.828; 10.037]
HF normalized power *	0.077	[−0.302; 0.394]	[−9.673; 13.001]	[−10.018; −6.818]	[4.459; 15.435]
Total power *	18.625	[7.175; 48.412]	[−1983.120; 159.941]	[−2346.200; −676.945]	[143.153; 161.655]
LF/HF *	−0.007	[−0.026; 0.007]	[−0.661; 0.371]	[−0.682; −0.583]	[0.174; 0.424]
Poincaré SD1 *	0.169	[−0.021; 0.285]	[−9.390; 3.067]	[−10.327; −5.012]	[2.320; 3.194]
Poincaré SD2 *	0.229	[0.083; 0.399]	[−11.718; 3.699]	[−13.841; −4.232]	[3.039; 3.741]
SD2/SD1 *	−0.001	[−0.012; 0.012]	[−0.126; 0.180]	[−0.133; −0.093]	[0.144; 0.189]
Approximate entropy	0	[−0.012; 0.013]	[−0.068; 0.068]	[−0.090; −0.046]	[0.046; 0.090]
Sample entropy *	0.002	[−0.036; 0.041]	[−0.128; 0.652]	[−0.132; −0.109]	[0.117; 0.806]
DFA α1 *	0.001	[−0.002; 0.008]	[−0.189; 0.093]	[−0.196; −0.151]	[0.067; 0.098]
DFA α2 *	0.002	[0; 0.006]	[−0.016; 0.118]	[−0.017; −0.009]	[0.084; 0.125]

* Non-normal distribution of differences.

**Table 4:** Results of correlation and Passing-Bablok linear regression analyses performed on GCG and ECG signals from the healthy subjects dataset (29 subjects).

*HRV Index*	*r*	*CI_r_*	*Slope*	*CI_slope_*	*Intercept*	*CI_intercept_*
Mean RR *	0.999	[0.997; 0.999]	1.000	[0.999; 1.001]	−0.044	[−0.526; 1.166]
SDNN *	0.995	[0.990; 0.998]	0.986	[0.955; 1.002]	0.983	[0.307; 2.255]
Mean HR *	0.999	[0.998; 1.000]	1.000	[0.999; 1.001]	−0.004	[−0.039; 0.079]
SD HR *	0.994	[0.986; 0.997]	0.993	[0.961; 1.007]	0.054	[0.008; 0.160]
Min HR *	0.994	[0.987; 0.997]	0.997	[0.986; 1.000]	0.137	[0.011; 0.793]
Max HR *	0.933	[0.861; 0.968]	1.001	[0.998; 1.007]	−0.089	[−0.517; 0.202]
RMSSD *	0.990	[0.979; 0.995]	0.975	[0.938; 0.995]	1.254	[0.388; 2.792]
NN50 *	0.996	[0.992; 0.998]	1.010	[0.992; 1.031]	0.651	[−0.653; 1.300]
pNN50 *	0.994	[0.988; 0.997]	1.009	[0.986; 1.026]	0.124	[−0.331; 0.865]
LF absolute power *	0.982	[0.962; 0.992]	1.013	[0.999; 1.031]	1.112	[−10.491; 13.718]
HF absolute power *	0.985	[0.968; 0.993]	1.017	[0.997; 1.038]	0.588	[−16.651; 16.293]
LF relative power *	0.966	[0.929; 0.984]	1.007	[0.988; 1.033]	−0.617	[−1.693; 0.331]
HF relative power *	0.976	[0.950; 0.989]	1.010	[0.996; 1.034]	−0.189	[−1.265; 0.522]
LF normalized power *	0.971	[0.939; 0.987]	1.008	[0.992; 1.032]	−0.684	[−1.791; 0.220]
HF normalized power *	0.971	[0.939; 0.987]	1.008	[0.992; 1.032]	−0.069	[−1.415; 0.616]
Total power *	0.990	[0.979; 0.995]	1.012	[0.996; 1.021]	4.421	[−9.378; 37.896]
LF/HF *	0.972	[0.940; 0.987]	0.996	[0.971; 1.024]	−0.008	[−0.030; 0.011]
Poincaré SD1 *	0.990	[0.979; 0.995]	0.975	[0.938; 0.995]	0.888	[0.273; 1.983]
Poincaré SD2 *	0.990	[0.978; 0.995]	0.995	[0.977; 1.008]	0.717	[0.019; 1.629]
SD2/SD1 *	0.965	[0.925; 0.983]	0.994	[0.939; 1.018]	0.010	[−0.030; 0.108]
Approximate entropy *	0.992	[0.982; 0.996]	0.997	[0.971; 1.037]	0.009	[−0.032; 0.034]
Sample entropy *	0.871	[0.740; 0.938]	0.962	[0.863; 1.106]	0.066	[−0.144; 0.221]
DFA α1 *	0.957	[0.909; 0.980]	1.015	[0.989; 1.074]	−0.018	[−0.065; 0.007]
DFA α2 *	0.974	[0.944; 0.988]	0.974	[0.942; 1.002]	0.005	[−0.001; 0.013]

* cusum linearity test *p*-value > 0.1.

**Table 5:** Results of Bland-Altman analysis performed on GCG and ECG signals from the healthy subjects dataset (29 subjects). In cases of measurement differences with a non-normal distribution, the bias was estimated as the median of differences and the limits of agreement as the 2.5th and 97.5th percentiles, respectively.

*HRV Index*	*Bias*	*CI_bias_*	*LoA*	*CI_LoA min_*	*CI_LoA max_*
Mean RR *	0.004	[−0.008; 0.034]	[−15.224; 21.364]	[−18.838; −2.367]	[9.774; 23.969]
SDNN *	0.405	[0.146; 0.541]	[−7.446; 5.486]	[−8.708; −2.482]	[4.146; 5.651]
Mean HR *	0	[−0.002; 0.001]	[−1.000; 0.995]	[−1.097; −0.090]	[0.134; 1.239]
SD HR *	0.031	[0.022; 0.046]	[−0.324; 0.448]	[−0.359; −0.168]	[0.354; 0.454]
Min HR *	−0.012	[−0.028; 0]	[−2.481; 2.030]	[−2.99; −0.700]	[1.533; 2.055]
Max HR *	0.019	[−0.021; 0.046]	[−12.138; 8.000]	[−13.549; −6.201]	[5.643; 8.366]
RMSSD *	0.199	[0.024; 0.583]	[−13.48; 1.884]	[−13.785; −11.135]	[1.715; 1.896]
NN50 *	1.000	[0; 3.000]	[−26.475; 9.325]	[−33.000; −3.325]	[6.775; 10.000]
pNN50 *	0.429	[0; 0.893]	[−7.631; 2.866]	[−8.674; −3.361]	[2.264; 2.941]
LF absolute power *	11.633	[3.469; 18.106]	[−1637.084; 886.249]	[−2051.11; −167.098]	[150.984; 1094.418]
HF absolute power *	14.505	[2.195; 41.863]	[−862.011; 262.361]	[−982.636; −349.895]	[195.484; 273.396]
LF relative power *	−0.267	[−0.585; 0.032]	[−4.241; 18.982]	[−4.702; −2.518]	[1.750; 23.901]
HF relative power *	0.368	[0.029; 0.584]	[−17.048; 2.344]	[−21.236; −2.147]	[2.069; 2.347]
LF normalized power *	−0.317	[−0.686; 0.002]	[−3.245; 18.754]	[−3.533; −2.132]	[2.088; 23.474]
HF normalized power *	0.317	[−0.001; 0.684]	[−18.744; 3.256]	[−23.463; −2.081]	[2.128; 3.549]
Total power *	25.093	[15.185; 58.74]	[−1830.18; 406.8]	[−2343.112; −51.739]	[304.384; 420.661]
LF/HF *	−0.011	[−0.023; 0]	[−0.230; 1.177]	[−0.239; −0.172]	[0.178; 1.456]
Poincaré SD1 *	0.141	[0.017; 0.412]	[−9.553; 1.335]	[−9.771; −8.047]	[1.247; 1.342]
Poincaré SD2 *	0.495	[0.318; 0.695]	[−7.662; 14.356]	[−9.463; −1.324]	[7.332; 15.873]
SD2/SD1 *	0.002	[−0.007; 0.007]	[−0.113; 0.520]	[−0.120; −0.076]	[0.238; 0.590]
Approximate entropy *	0.005	[−0.007; 0.013]	[−0.086; 0.101]	[−0.101; −0.030]	[0.066; 0.108]
Sample entropy *	0.005	[−0.009; 0.021]	[−0.258; 0.485]	[−0.278; −0.163]	[0.374; 0.490]
DFA α1 *	−0.003	[−0.010; 0]	[−0.073; 0.315]	[−0.079; −0.050]	[0.116; 0.369]
DFA α2 *	0	[−0.001; 0.002]	[−0.043; 0.093]	[−0.048; −0.021]	[0.074; 0.097]

* Non-normal distribution of differences.

**Table 6:** Results of correlation and Passing-Bablok linear regression analyses performed on SCG and ECG signals from the pathological subjects dataset (42 subjects).

*HRV Index*	*r*	*CI_r_*	*Slope*	*CI_slope_*	*Intercept*	*CI_intercept_*
Mean RR *	0.9999	[0.9999; 1.0000]	1.002	[1.000; 1.005]	−1.309	[−3.618; −0.044]
SDNN *	0.9944	[0.9895; 0.9970]	0.967	[0.939; 0.997]	0.423	[0.063; 0.942]
Mean HR *	0.99995	[0.9999; 1.0000]	1.001	[1.000; 1.004]	−0.115	[−0.336; −0.024]
SD HR *	0.9911	[0.9835; 0.9953]	0.971	[0.947; 1.006]	0.045	[−0.002; 0.075]
Min HR *	0.9990	[0.9982; 0.9995]	0.999	[0.997; 1.001]	0.026	[−0.082; 0.166]
Max HR **	0.9988	[0.9977; 0.9993]	1.005	[1.001; 1.016]	−0.354	[−1.261; −0.033]
RMSSD *	0.9960	[0.9925; 0.9978]	0.945	[0.907; 0.978]	0.790	[0.093; 1.349]
NN50 *	0.9668	[0.9388; 0.9822]	0.939	[0.826; 0.980]	0	[0; 0]
pNN50 *	0.9797	[0.9623; 0.9891]	0.939	[0.812; 0.988]	0	[0; 0]
LF absolute power *	0.9665	[0.9382; 0.9820]	1.034	[0.994; 1.084]	0.013	[−1.461; 0.983]
HF absolute power *	0.9994	[0.9988; 0.9997]	0.921	[0.859; 0.947]	2.071	[0.118; 7.406]
LF relative power *	0.9011	[0.8223; 0.9459]	0.991	[0.872; 1.094]	1.522	[−2.258; 6.836]
HF relative power *	0.9401	[0.8907; 0.9676]	0.970	[0.871; 1.072]	−0.034	[−5.927; 4.012]
LF normalized power *	0.9278	[0.8688; 0.9608]	0.988	[0.884; 1.103]	1.789	[−2.565; 7.255]
HF normalized power *	0.9279	[0.8691; 0.9609]	0.989	[0.883; 1.104]	−0.630	[−7.780; 4.439]
Total power *	0.9946	[0.9900; 0.9971]	0.975	[0.938; 1.029]	5.212	[−2.606; 12.354]
LF/HF *	0.8127	[0.6757; 0.8955]	1.043	[0.923; 1.197]	0.013	[−0.038; 0.097]
Poincaré SD1 *	0.9960	[0.9925; 0.9978]	0.944	[0.906; 0.977]	0.566	[0.076; 0.964]
Poincaré SD2 *	0.9872	[0.9761; 0.9931]	0.973	[0.945; 0.996]	0.583	[0.061; 1.077]
SD2/SD1 *	0.9131	[0.8432; 0.9527]	0.936	[0.827; 1.074]	0.102	[−0.064; 0.252]
Approximate entropy *	0.952	[0.9119; 0.9741]	1.160	[1.029; 1.294]	−0.213	[−0.375; −0.047]
Sample entropy *	0.7910	[0.6413; 0.8827]	0.928	[0.799; 1.081]	0.107	[−0.170; 0.339]
DFA α1 *	0.9356	[0.8827; 0.9651]	0.969	[0.874; 1.080]	0.036	[−0.034; 0.127]
DFA α2 *	0.9535	[0.9146; 0.9749]	1.013	[0.951; 1.091]	−0.011	[−0.046; 0.016]

* cusum linearity test *p*-value > 0.1. ** cusum linearity test *p*-value > 0.05.

**Table 7:** Results of Bland-Altman analysis performed on SCG and ECG signals from the pathological subjects dataset (42 subjects). In cases of measurement differences with a non-normal distribution, the bias was estimated as the median of differences, and the limits of agreement as the 2.5th and 97.5th percentiles, respectively.

*HRV Index*	*Bias*	*CI_bias_*	*LoA*	*CI_LoA min_*	*CI_LoA max_*
Mean RR *	0.234	[0.031; 0.402]	[−2.045; 6.132]	[−2.990; −1.010]	[3.606; 8.2]
SDNN *	0.040	[−0.192; 0.352]	[−4.141; 4.414]	[−4.975; −2.941]	[2.889; 5.17]
Mean HR *	−0.014	[−0.037; −0.003]	[−0.381; 0.313]	[−0.505; −0.260]	[0.085; 0.556]
SD HR *	0.003	[−0.018; 0.031]	[−0.327; 0.343]	[−0.339; −0.241]	[0.279; 0.376]
Min HR *	−0.029	[−0.042; −0.013]	[−2.200; 0.511]	[−2.540; −1.472]	[0.099; 0.936]
Max HR *	0	[−0.023; 0.047]	[−2.227; 1.297]	[−2.823; −1.543]	[1.052; 1.423]
RMSSD *	−0.301	[−0.800; 0.456]	[−6.267; 2.886]	[−7.064; −4.406]	[2.718; 3.07]
NN50 *	−0.500	[−1.000; 0]	[−34.100; 6.900]	[−66.000; −7.000]	[5; 8]
pNN50 *	−0.074	[−0.196; 0]	[−9.148; 2.001]	[−16.561; −2.166]	[1.39; 2.103]
LF absolute power *	0.744	[0.122; 3.916]	[−124.02; 415.754]	[−151.708; −49.850]	[223.816; 615.848]
HF absolute power *	−2.725	[−9.616; −0.538]	[−581.508; 34.352]	[−1179.716; −87.789]	[27.523; 39.36]
LF relative power *	0.962	[−0.230; 3.585]	[−10.183; 26.251]	[−11.033; −7.283]	[21.037; 28.539]
HF relative power *	−1.345	[−4.163; 0.014]	[−24.080; 8.762]	[−26.460; −18.815]	[6.325; 11.33]
LF normalized power *	1.146	[−0.251; 4.520]	[−8.879; 26.748]	[−10.577; −7.105]	[19.137; 28.597]
HF normalized power *	−1.158	[−4.548; 0.128]	[−26.661; 8.886]	[−28.542; −18.413]	[7.1; 10.569]
Total power *	0.909	[−2.803; 6.909]	[−524.008; 374.227]	[−881.252; −163.948]	[152.056; 493.838]
LF/HF *	0.064	[−0.003; 0.220]	[−0.874; 2.623]	[−1.100; −0.616]	[0.921; 4.133]
Poincaré SD1 *	−0.212	[−0.566; 0.323]	[−4.436; 2.046]	[−4.999; −3.104]	[1.884; 2.18]
Poincaré SD2 *	0.007	[−0.156; 0.325]	[−5.381; 9.105]	[−6.320; −3.318]	[6.109; 12.683]
SD2/SD1 *	0.033	[−0.028; 0.082]	[−0.406; 0.570]	[−0.546; −0.241]	[0.434; 0.626]
Approximate entropy	−0.014	[−0.034; 0.006]	[−0.142; 0.115]	[−0.177; −0.108]	[0.08; 0.149]
Sample entropy *	−0.029	[−0.059; 0.017]	[−0.641; 0.314]	[−1.016; −0.311]	[0.206; 0.335]
DFA α1	0.028	[−0.005; 0.062]	[−0.192; 0.249]	[−0.252; −0.133]	[0.19; 0.308]
DFA α2 *	−0.006	[−0.014; 0.003]	[−0.096; 0.126]	[−0.098; −0.083]	[0.069; 0.17]

* Non-normal distribution of differences.

**Table 8:** Results of correlation and Passing-Bablok linear regression analyses performed on GCG and ECG signals from the pathological subjects dataset (49 subjects).

*HRV Index*	*r*	*CI_r_*	*Slope*	*CI_slope_*	*Intercept*	*CI_intercept_*
Mean RR *	0.9988	[0.9979; 0.9993]	1.001	[1.000; 1.003]	−0.611	[−1.974; 0.182]
SDNN *	0.9973	[0.9952; 0.9985]	0.958	[0.933; 0.981]	0.660	[0.290; 1.018]
Mean HR *	0.9993	[0.9988; 0.9996]	1.001	[1.000; 1.002]	−0.049	[−0.148; 0.015]
SD HR *	0.9926	[0.9868; 0.9958]	0.953	[0.922; 0.991]	0.057	[0.007; 0.101]
Min HR *	0.9939	[0.9892; 0.9966]	1.000	[0.996; 1.003]	−0.026	[−0.200; 0.225]
Max HR *	0.9697	[0.9465; 0.9829]	1.002	[0.999; 1.005]	−0.117	[−0.346; 0.084]
RMSSD *	0.9655	[0.9393; 0.9805]	0.905	[0.861; 0.956]	1.064	[0.304; 1.858]
NN50 *	0.9480	[0.9092; 0.9705]	0.887	[0.722; 1.000]	0	[0; 0]
pNN50 *	0.9619	[0.9331; 0.9785]	0.890	[0.755; 1.000]	0	[0; 0]
LF absolute power *	0.8218	[0.7031; 0.8960]	1.017	[0.994; 1.061]	0.534	[−0.483; 2.067]
HF absolute power *	0.9264	[0.8724; 0.9580]	0.854	[0.772; 0.921]	2.643	[0.118; 7.785]
LF relative power *	0.8712	[0.7815; 0.9257]	0.996	[0.913; 1.070]	1.794	[−1.364; 5.745]
HF relative power *	0.8469	[0.7426; 0.9111]	0.994	[0.895; 1.073]	−1.396	[−4.919; 2.196]
LF normalized power *	0.8567	[0.7581; 0.9170]	0.991	[0.900; 1.069]	2.662	[−1.178; 8.195]
HF normalized power *	0.8571	[0.7587; 0.9172]	0.991	[0.899; 1.069]	−1.910	[−5.770; 1.857]
Total power *	0.9792	[0.9632; 0.9883]	0.945	[0.891; 0.983]	6.346	[2.552; 14.176]
LF/HF *	0.8069	[0.6800; 0.8869]	1.107	[0.965; 1.220]	−0.004	[−0.088; 0.141]
Poincaré SD1 *	0.9655	[0.9393; 0.9805]	0.905	[0.861; 0.956]	0.754	[0.216; 1.314]
Poincaré SD2 *	0.9879	[0.9785; 0.9932]	0.975	[0.950; 1.005]	0.516	[−0.101; 0.871]
SD2/SD1 *	0.8623	[0.7671; 0.9203]	0.952	[0.847; 1.037]	0.109	[−0.012; 0.270]
Approximate entropy *	0.9353	[0.8875; 0.9632]	0.989	[0.896; 1.129]	0.003	[−0.162; 0.107]
Sample entropy *	0.7906	[0.6551; 0.8769]	0.884	[0.754; 1.092]	0.166	[−0.160; 0.414]
DFA α1 *	0.8812	[0.7976; 0.9315]	0.943	[0.830; 1.054]	0.078	[−0.026; 0.160]
DFA α2 *	0.976	[0.9576; 0.9864]	0.919	[0.869; 0.968]	0.026	[0.008; 0.048]

* cusum linearity test *p*-value > 0.1.

**Table 9:** Results of Bland-Altman analysis performed on GCG and ECG signals from the pathological subjects dataset (49 subjects). In cases of measurement differences with a non-normal distribution, the bias was estimated as the median of differences and the limits of agreement as the 2.5th and 97.5th percentiles, respectively.

*HRV Index*	*Bias*	*CI_bias_*	*LoA*	*CI_LoA min_*	*CI_LoA max_*
Mean RR *	0.089	[0.017; 0.326]	[−13.877; 9.309]	[−44.947; −1.722]	[3.849; 22.720]
SDNN *	−0.072	[−0.443; 0.175]	[−4.526; 3.22]	[−5.308; −3.330]	[1.852; 5.051]
Mean HR *	−0.007	[−0.031; −0.002]	[−0.541; 0.94]	[−1.199; −0.236]	[0.187; 2.923]
SD HR *	−0.011	[−0.029; 0.010]	[−0.439; 0.379]	[−0.703; −0.300]	[0.180; 0.475]
Min HR *	−0.026	[−0.046; 0]	[−3.967; 0.782]	[−9.512; −1.688]	[0.215; 1.992]
Max HR *	0.032	[0; 0.060]	[−0.875; 14.57]	[−1.057; −0.440]	[4.855; 18.485]
RMSSD *	−0.409	[−1.223; 0.059]	[−33.607; 4.577]	[−62.82;0 −12.495]	[3.774; 6.171]
NN50 *	0	[−1.000; 0]	[−72.8; 9.1]	[−96.000; −24.125]	[4.750; 12.000]
pNN50 *	0	[−0.159; 0]	[−20.793; 2.74]	[−25.810; −7.614]	[1.673; 2.793]
LF absolute power *	1.507	[0.859; 5.145]	[−95.543; 2286.372]	[−132.437; −44.195]	[537.339; 6673.552]
HF absolute power *	−5.075	[−23.853; −0.293]	[−2952.447; 80.261]	[−5421.979; −793.827]	[33.796; 100.829]
LF relative power *	1.562	[0.069; 3.266]	[−6.02; 40.733]	[−6.082; −5.184]	[23.365; 50.334]
HF relative power *	−1.568	[−3.584; −0.135]	[−50.884; 8.237]	[−60.051; −30.322]	[6.946; 10.86]
LF normalized power *	2.151	[0.156; 3.752]	[−8.319; 47.927]	[−10.683; −6.871]	[30.824; 58.034]
HF normalized power *	−2.276	[−3.770; −0.040]	[−47.763; 8.307]	[−57.968; −30.719]	[7.242; 10.758]
Total power *	0.318	[−22.003; 5.581]	[−604.787; 1136.585]	[−1414.523; −255.895]	[201.45; 2473.06]
LF/HF *	0.109	[0.001; 0.190]	[−1.572; 5.298]	[−2.751; −1.019]	[2.435; 11.744]
Poincaré SD1 *	−0.289	[−0.866; 0.042]	[−23.799; 3.241]	[−44.49; −7.980]	[2.672; 4.370]
Poincaré SD2 *	0.019	[−0.200; 0.304]	[−3.916; 16.183]	[−4.601; −2.846]	[6.266; 24.955]
SD2/SD1 *	0.048	[0.010; 0.086]	[−0.689; 1.024]	[−0.848; −0.403]	[0.728; 1.145]
Approximate entropy	−0.016	[−0.035; 0.002]	[−0.147; 0.114]	[−0.179; −0.115]	[0.082; 0.147]
Sample entropy *	−0.005	[−0.057; 0.032]	[−0.572; 0.409]	[−0.608; −0.368]	[0.264; 0.411]
DFA α1 *	0.025	[−0.016; 0.053]	[−0.230; 0.545]	[−0.244; −0.180]	[0.339; 0.599]
DFA α2 *	−0.003	[−0.017; 0.001]	[−0.14; 0.081]	[−0.169; −0.079]	[0.049; 0.122]

* Non-normal distribution of differences.

## Data Availability

All relevant research data will be made available upon request after the publication of the paper.

## Appendix A

**Table A1:** Patient ID# of the pathological subjects considered for HRV analyses on SCG and GCG signals.

Patient ID#	
SCG	GCG
CP01	CP01
CP02	CP02
CP05	CP05
**Not Included**	CP06
CP07	CP07
CP08	CP08
CP13	CP13
CP15	CP15
**Not Included**	CP17
**Not Included**	CP18
CP19	CP19
CP20	CP20
CP21	CP21
CP23	CP23
CP27	CP27
CP28	CP28
CP30	CP30
CP34	CP34
CP36	CP36
**Not Included**	CP37
CP39	CP39
CP41	CP41
CP44	CP44
CP45	CP45
**Not Included**	CP50
**Not Included**	CP51
CP53	CP53
CP57	CP57
CP58	CP58
CP59	CP59
CP60	CP60
CP61	CP61
CP63	CP63
CP65	CP65
CP66	CP66
CP69	CP69
UP01	**Not Included**
UP02	UP02
UP04	UP04
UP07	UP07
UP08	UP08
UP09	UP09
UP11	UP11
UP12	UP12
UP14	UP14
UP15	UP15
UP17	**Not Included**
UP20	UP20
UP26	UP26
UP29	UP29
UP30	UP30
